# The Relationship between Physical Activity and Bone during Adolescence Differs according to Sex and Biological Maturity

**DOI:** 10.4061/2010/546593

**Published:** 2010-09-20

**Authors:** Benjamin K. Weeks, Belinda R. Beck

**Affiliations:** ^1^Griffith Health Institute, Griffith University, QLD 4222, Australia; ^2^School of Physiotherapy and Exercise Science, Gold Coast Campus, Griffith University, QLD 4222, Australia

## Abstract

This study examines the relationships between bone mass, physical activity, and maturational status in healthy adolescent boys and girls. *Methods*. Ninety-nine early high-school (Year 9) students were recruited. Physical activity and other lifestyle habits were recorded via questionnaire. Anthropometrics, muscle power, calcaneal broadband ultrasound attenuation (BUA), bone mineral content (BMC), and lean tissue mass were measured. Maturity was determined by Tanner stage and estimated age of peak height velocity (APHV). *Results*. Boys had greater APHV, weight, height, muscle power, and dietary calcium than girls (*P* < .05). Boys exhibited greater femoral neck BMC and trochanteric BMC while girls had higher BUA and spine BMAD (*P* < .05). Physical activity and vertical jump predicted BMAD and BUA most strongly for boys whereas years from APHV were the strongest predictor for girls. *Conclusion*. Sex-specific relationships exist between physical activity, maturity and bone mass during adolescence.

## 1. Introduction

Bone mass increases during growth to reach a peak in young adulthood, plateaus, and declines thereafter. Maximizing the peak bone mass achieved and maintaining bone strength throughout life may be the most effective strategies to reduce the risk of fracture in the later years of life. Genetics, maturity and hormone status, nutrition, muscle force, and physical activity are known to influence bone mineral accrual during childhood; however, the relative extent of their contribution for boys and girls during adolescence is poorly understood. 

Factors such as physical activity have shown strong associations to bone mass in children [1–3], often in a sex-specific fashion [2, 3]. For instance, moderate-to-vigorous activity has been observed to be strongly associated with lower-limb bone mineral density (BMD) and bone mineral content (BMC), but it was only associated with total body bone mass in young (11-year-old) boys [3]. Similarly, high-impact physical activity in older boys (16-to-18 years old) shows strong positive relationships to whole body BMC and total hip BMC [1]. In a recent cross-sectional study of BMC and volumetric BMD (vBMD) across the lifespan (8-to-85 years old), physical activity was positively associated with femoral neck BMC in men, but not women, while physical activity was more strongly associated with cortical vBMD at the radius for women than for men. Additionally, sex differences (in favor of boys) in the development of bone strength at the femoral neck during the adolescent growth spurt have been observed [4]. Should the factors that influence bone development during adolescence differ according to sex, it would be appropriate to customize bone-relevant interventions accordingly.

Maximizing skeletal exposure to mechanical loading (physical activity) during growth appears to be an effective strategy to optimize bone accrual [5]. Animal studies of the bone response to mechanical loads have revealed that loads imposing high-magnitude strains [6] at high strain rates [7] result in greater osteogenic response than those associated with low magnitudes and rates. In order to translate the findings of animal research into useful practical application, physical activities that effectively impart osteogenic loads to the growing skeleton must be identified. Examining the effect of limited skeletal loading, as a consequence of chronic physical inactivity (e.g., television watching and computer use), on bone development is similarly vital.

The transition through puberty is accompanied by increasing levels of circulating sex steroids, peaking levels of circulating growth hormone and insulin-like growth factor-1 (IGF-1), and associated peak rates of bone growth. Hormones known to enhance bone formation decrease after puberty [8–10]. For example, the reduction in IGF-1 following menarche may reduce skeletal sensitivity to mechanical loading [11]. Maturational status is therefore a critical consideration when examining the effect of physical activity on the adolescent skeleton. 

The chronological age of children of the same maturational status can vary widely. Historically, Tanner stages [12] determined by self-assessment or visual inspection by a third party have been used to classify children according to physical and sexual maturity. Although Tanner staging via visual inspection might be appropriate for clinical studies, its use in the study of healthy populations, such as for exercise interventions, can be less acceptable. Mirwald and colleagues [13] recently developed a considerably less confronting method of determining maturational status, which involves the estimation of age of peak height velocity (APHV). The calculation is based on chronological age and anthropometric ratios of weight, standing height, and sitting height and has been validated [13] with data collected in a large longitudinal pediatric trial [14]. APHV may be particularly applicable to the determination of biological maturity. 

The primary aim of the current investigation was to identify potential sex differences in the relationships between physical activity and maturational status to bone mass and quality during the adolescent years.

## 2. Methods

### 2.1. Subjects and Subject Selection

A total of 99 Caucasian adolescents (46 boys mean age, 13.8 ± 0.4 years; 53 girls mean age 13.7 ± 0.4  years) enrolled in the ninth grade of a local state high school (Pacific Pines, Gold Coast, Australia) were recruited on a volunteer basis. Subjects were included if in sound general health and fully ambulatory. Subjects were excluded if they had a metabolic bone disease, endocrine disorder, or chronic renal pathology; were taking medications known to affect bone; were recovering from lower-limb fracture or other immobilised injury; or were affected by any condition not compatible with performing routine physical activity. Ethical approval was obtained from the Griffith University Human Research Ethics Committee and Education Queensland (Queensland Government Department for Education, Training and the Arts). Written informed consent was obtained from the participants and their parents or guardians.

### 2.2. Subject Measurement

Subject measures included anthropometrics, assessment of, maturity, and evaluation of muscle, bone, physical activity and diet.

#### 2.2.1. Anthropometrics

Subject height and sitting height were measured to the nearest millimetre using the stretch stature method with a portable stadiometer (HART Sport & Leisure, Australia). Weight was measured to the nearest 0.1 kilogram using the mean of measures from two sets of digital scales (Soehnle Co., Switzerland). Body mass index (BMI) was determined from measures of height (m) and weight (kg) and calculated as BMI = weight·height^−2^.

#### 2.2.2. Assessment of Maturity

Maturity was determined using two methods. Subjects were asked to self-determine Tanner stage using standard diagrams of pubic hair growth and breast or penis/scrotum development [12]. If responses for the two categories differed, then pubic hair stage was used to define Tanner stage. Privacy was maintained from other subjects and investigators by providing booths for completing forms and placing them in sealed, coded envelopes for later analysis. In addition, the method of Mirwald and colleagues [13] was used to predict years from age at PHV (YAPHV) based on single measures of height, sitting height, body mass, and chronological age.

#### 2.2.3. Muscle Power

Muscle power was estimated using a vertical jump test. The Yardstick (Swift Sports Equipment, Lismore, Australia) was used to measure vertical jump height as the difference between the height of a standing reach and maximum absolute jump height. The subject stood with feet shoulder-width apart, preferred arm raised, and nonpreferred arm kept to the side of the body. A jump for maximum height was made in a countermovement fashion without armswing as per the protocol used by Young and colleagues [15]. The best of three attempts was recorded to the nearest centimetre.

#### 2.2.4. Bone Measures

The QUS-2 Ultrasound Densitometer (Quidel Corporation, Mountain View, CA, USA) was used to evaluate broadband ultrasound attenuation (BUA) of the nondominant foot. BUA has been shown to predict vertebral and proximal femoral fracture risk [16, 17] and discriminate between men with and without fractures [18]. The same investigator (B.R.Beck) performed all ultrasound assessments. Calibration quality control was accomplished via an automated verification process that involved the scanning of a phantom model of known BUA each day of testing. Repeat scans in this cohort (*n* = 20) with repositioning determined short-term BUA measurement precision (CV) of 2.8%.

Measures of bone mineral content (BMC), bone mineral density (BMD), and bone area (BA) of the nondominant femoral neck (FN) and trochanter (TR), lumbar spine (LS), and whole body (WB) were made with an XR-36 Quickscan Densitometer (Norland Medical Systems, Inc., USA) using host software, Version 2.5.3a. Bone mineral apparent density (BMAD) was calculated as a means of size-correcting BMD as recommended by Fewtrell and colleagues [19]. Mechanical characteristics including LS index of bone structural strength (IBS) and FN cross-sectional moment of inertia (CSMI) were derived from DXA measures using formulae described by Sievänen and colleagues [20]. Measures of lean mass, fat mass, and body fat percentage (Siri method) were taken from WB scans. The same investigator (B.K.Weeks) performed and analyzed all DXA measurements. Short-term precision for repeated measures with repositioning on a subsample of the cohort (*n* = 35) for FN, LS, and WB BMC was 1.3%, 1.1%, and 1.4%, respectively.

#### 2.2.5. Physical Activity

Subjects completed a bone-specific physical activity questionnaire (BPAQ) designed to record past and current (previous 12 months) physical activities. Questions relating to sedentary activities (e.g., television watching and computer use) were also asked. Based on the relevance of load magnitude, rate, and frequency of application to bone adaptation [21], a method of calculating an index of bone-relevant weight-bearing exercise history for each subject was formulated. An algorithm, developed as a program on LabVIEW software (National Instruments, Texas, USA), was run for every subject in order to quantify historical physical activity-related bone loading for each individual using information provided in the BPAQ, incorporating weighting factors for impact intensity, frequency of exercise bouts, and years of participation of each type of exercise. 

The BPAQ and bone loading algorithm were specifically developed and validated for use in the bone research investigations of our group. BPAQ validation included force platform testing to verify impact ratings and cross-validation of BPAQ scores with scores derived from other recognized Physical Activity measurement instruments [22–24]. Scores from the BPAQ have been shown to predict variance (up to 60%) in indices of bone strength at the FN and LS [25].

#### 2.2.6. Calcium Intake

Dietary calcium consumption was estimated from a calcium-focused food questionnaire. Subjects were asked to indicate the type and amount of each food item they consume. The average daily intake of dietary calcium was calculated using *Calcium Calculator*, an internet-based java applet program obtained from *CalciumInfo.com* [26] that uses a database of calcium values for common foods.

### 2.3. Statistical Analyses

One-way ANOVA was used to examine gender differences in subject characteristics. ANCOVA (general linear model) was used to examine gender differences in bone parameters with height and weight serving as covariates. APHV (biological maturity) and BPAQ score (physical activity) were additionally included as covariates in separate analyses. Multiple regression analysis with independent variables entered in forward stepwise fashion was employed to investigate the influence of physical activity, lifestyle, and dietary factors, including covariate analyses to control for factors such as height, weight, and maturity. Two-tailed Pearson correlations were employed to observe relationships between lifestyle factors, such as physical activity and calcium intake and regional bone parameters. Statistical significance was set at *P* < .05. All statistical analyses were performed using SPSS version 15.0 for Windows (SPSS, Chicago, IL, USA).

## 3. Results

### 3.1. Subject Characteristics

Subject characteristics are summarised in Table 1. Ninety-nine volunteers (46 boys and 53 girls) participated in the study. Boys were heavier, taller, and had greater vertical jump performance than girls (*P* < .05). Boys had significantly greater lean mass (22%) and lower percent body fat (5%) than girls (*P* < .002). Boys consumed more dietary calcium (38%) than girls (*P* = .004). There were no sex differences in bone-specific physical activity questionnaire (BPAQ) score or time spent watching television. 

Boys recorded a significantly older APHV (13.8 ± 0.1 years) than girls (12.3 ± 0.1 years). As male and female volunteers were of similar age, males had significantly fewer years to APHV (0.0 ± 0.1 years) than females (1.5 ± 0.1 years). All Tanner stages were represented in both boys and girls; however, most (53%) were Tanner IV. Tanner stage and YAPHV were positively correlated for both boys (*r* = 0.461; *P* = .001) and girls (*r* = 0.356; *P* = .006), however, unlike APHV and YAPHV, no sex difference in maturity could be detected using Tanner stage. Average age of menarche for girls in the study cohort was 12.5 ± 0.7 with 11 being premenarcheal and 42 being postmenarcheal at the time of testing. 

### 3.2. Bone Parameters

Height and weight were significant predictors of all bone parameters for both boys and girls (*P* < .05); thus, indices of bone strength were first investigated with only height and weight included as covariates. Girls had greater BUA than boys (*P* = .03). Boys exhibited more substantial proximal femora than girls (greater FN area, FN BMC, FN CSMI, TR BMC, and TR BMD, *P* ≤ .01) while girls had more robust lumbar spines than boys (greater LS BMD, LS BMAD, and LS IBS, *P* ≤ .01). No sex differences were evident for WB measures of bone mass. 

When APHV was adjusted for, boys exhibited greater WB BMC (*P* = .001) and WB BMD (*P* = .001), differences at the calcaneus were lost (*P* = .542), and the significance of greater measurements for boys for FN BMC and FN BMD was strengthened (*P* = .001). When physical activity (BPAQ score) was adjusted for, statistical differences remained unchanged; however, when vertical jump height was additionally controlled for, the significance of greater FN measures in boys than girls disappeared. Table 2 displays results for bone parameters for both sexes. 

**Figure 1:**
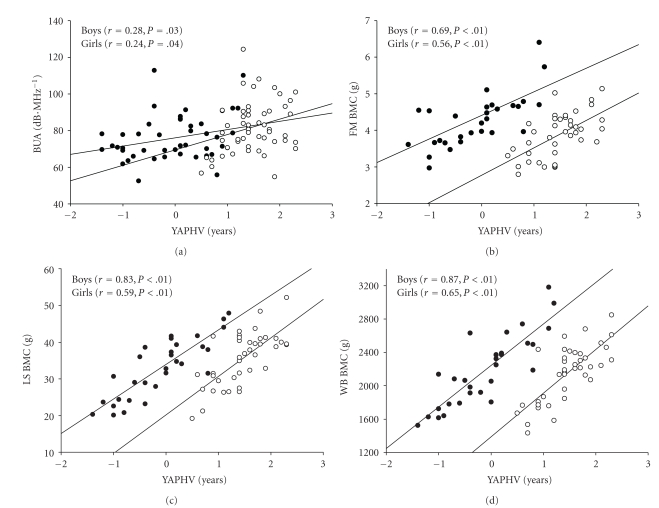
Correlation plots showing relationships between bone parameters and years from peak height velocity (YAPHV) for boys (closed circles) and girls (open circles). (a) BUA, broadband ultrasound attenuation; (b) FN BMC, femoral neck bone mineral content; (c) LS BMC, lumbar spine bone mineral content; and (d) WB BMC, whole-body bone mineral content.

**Figure 2:**
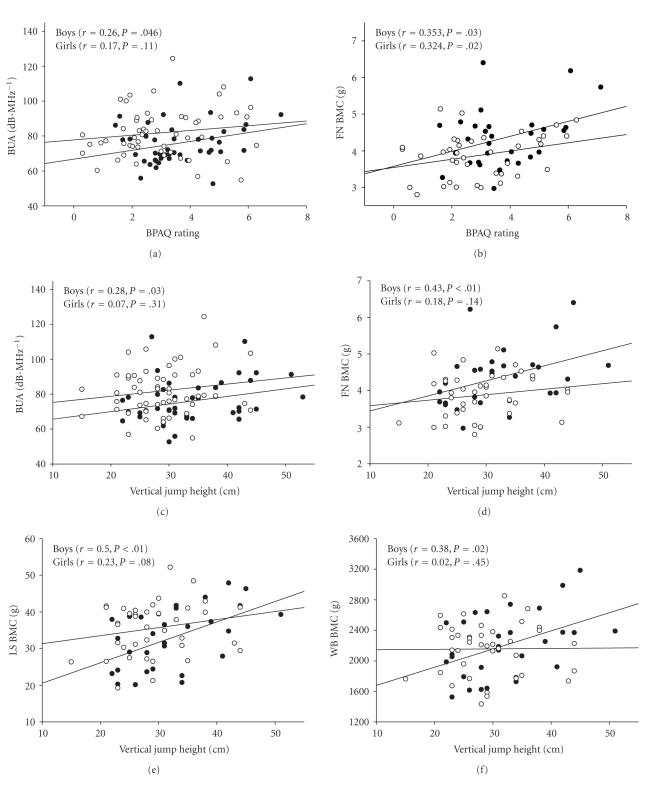
Correlation plots showing relationships between bone parameters and bone-specific physical activity questionnaire (BPAQ) score and vertical jump height for boys (closed circles) and girls (open circles). (a) BUA, broadband ultrasound attenuation; (b) FN BMC, femoral neck bone mineral content; (c) BUA, broadband ultrasound attenuation; (d) FN BMC, femoral neck bone mineral content; (e) LS BMC, lumbar spine bone mineral content; and (f) WB BMC, whole-body bone mineral content.

YAPHV, BPAQ score, and vertical jump height were all found to account for variance in bone parameters. YAPHV was a significant predictor of the majority of bone measures in girls (FN BMAD, FN CSMI, LS BMC, LS BMAD, LS IBS, TR BMC, and WB BMC), but it accounted for variance in only trochanteric BMC for boys (Figures 1(a)–1(d)). BPAQ score predicted a large proportion of bone measures for boys (BUA, FN BMC, FN CSMI, LS BMC, LS IBS, TR BMC, and WB BMC), but only FN BMC, FN BMAD, and TR BMC for girls (Figures 2(a)-2(b)). Vertical jump height was similarly predictive of bone strength parameters at multiple sites in boys (FN BMAD, FN CSMI, LS BMAD, LS IBS, TR BMC, and WB BMC), but predictive of only FN BMAD, LS BMC, and LS BMAD in girls (Figures 2(c)–2(f)). 

Daily dietary calcium intake was not found to predict parameters of bone strength. Time spent using a computer or sending text messages on a mobile phone showed no significant relationships with bone parameters for either sex; however, time spent watching television consistently showed significant inverse relationships with BPAQ score (*r* = −0.37; *P* = .009), and many of the bone parameters (*r* = −0.44 to −0.35; *P* < .05) (Table 3). 

## 4. Discussion

While others have reported that sex differences exist in the bone status of children [2, 27–29], our data extends these findings specifically to the peripubertal adolescent, and identifies sex-specific regional differences in parameters of bone strength. Physical activity and muscle power were the strongest predictors of bone mass in boys, while biological maturity best predicted bone mass in girls. In addition, we observed that television watching was inversely related to the parameters of bone strength for both sexes in this age group.

We found that, in Caucasian children aging 13-14 years, girls had stronger bone at the lumbar spine and heel than boys, while the reverse was true at the hip. The observed sex differences were attributable to differences in biological maturity and levels of physical activity. For girls, maturity accounted for variance in most bone parameters, including hip and spine bone mass, while physical activity could account for variance (10%) only at the hip. The tendency for greater bone mass at the spine and calcaneus in girls compared with chronological age-matched boys disappeared when age at PHV was controlled for; however, whole-body and hip bone mass remained greater in boys than girls. Greater bone mass at femoral sites in boys compared with chronological age-matched girls was related to level of physical activity. Controlling for vertical jump height removed the sex difference at the hip, corroborating the common theory that local intense muscle loading is an effective mechanical stimulus for bone.

The lack of relationship between bone mass and physical activity in the predominantly postmenarcheal girls of our cohort suggests either that factors determining overall physical growth and maturity have the strongest influence on female bone at this age or that the skeleton has become less responsive to physical activity. It has been observed that hormone factors that enhance bone formation decrease following attainment of PHV [8–10]. MacKelvie and others [11] postulated that the reduction in the concentration of growth hormone and insulin-like growth factor-1 (IGF-1) following menarche might explain a less mechanically sensitive skeleton in girls at this age. Wang and colleagues [30] similarly reported that the maturational status of early pubertal Finnish girls (10–12 years old) accounted for more of the variation in bone mass than physical activity history. That the girls in the Finnish study were approximately two years younger than those in the current Australian study (mean = 13.7 years) suggests that the effect of maturation, or minimal effect of physical activity, may be evident in girls from a relatively young age (at least 10 years old) and endure through puberty. Only investigations of girls older than those measured in either study will ascertain whether the effect is sustained or reverses with age. 

For boys, indices of bone strength, such as cross-sectional moment of inertia (a value that reflects strength as a function of resistance to bending), were most strongly associated with measures of physical activity. Given the relationship of muscle power to bone mass in boys, it is likely that muscle force accounts for this association [31]. Similar findings were reported by Macdonald and colleagues [28] who found that geometric measures of the tibia (using pQCT) in pre- and early pubertal children (aged 9–11) were significantly and positively correlated with physical activity in boys, but not girls, and with maturity in girls, but not boys. Girls in our study cohort were approximately 1.5 years past APHV, while the boys had just attained PHV. Increasing levels of circulating sex steroids and other growth factors may have conferred greater skeletal sensitivity to the mechanical loading in boys, compared with chronological age-matched girls. 

As developmental differences in the growth rates of the axial and appendicular skeleton have previously been observed [8, 32], maturation effects may be site specific and potentially account for differences in physical activity effect between skeletal locations. Growth of the appendicular skeleton is more rapid than that of the axial skeleton prior to puberty, while axial growth dominates bone development during the peripubertal years [32]. A two-year longitudinal study of girls spanning the ages of 7–17 years found that by age 11 approximately 40% of bone mineral had been accrued at the legs, while only 24% had accrued at the spine [8]. Given the lack of sex differences in LS BMD when APHV was controlled for, we suggest that the more advanced biological maturity of girls compared to boys of similar chronological age accounts for the greater lumbar spine bone mass in girls. Furthermore, as vertical mechanical loads are more greatly attenuated at the spine than the hip, the effects of physical activity could be expected to be less marked at the spine than at the hip. 

Despite larger anthropometric values, boys exhibited a lower calcaneal BUA than girls. As the reverse is the case in adults, it suggests an effect of differing rates of maturation of the predominantly trabecular calcaneus in boys compared with girls. Bone mineral accumulation during puberty is known to lag behind growth in bone size [33, 34]. It is possible that the lower bone mass at the spine and calcaneus observed in boys from the current cohort can be explained by less complete mineralization owing to larger bone size and less-advanced maturity than the girls. 

Our data clearly demonstrate significant negative relationships between time spent watching television and most parameters of bone strength in boys. Similar trends were observed for girls, although not all reached statistical significance. Our results may indicate that television watching displaces physical activity in this cohort, such that optimum mechanical loading of the skeleton is denied. This appears to be particularly important for boys in the current cohort. 

It is recognized that cross-sectional studies contain inherent design limitations. Furthermore, for logistical reasons it was necessary to rely on indirect measures of some variables, including physical activity, bone geometry, and dietary calcium. The limitations of data obtained from physical activity questionnaires are well known. Standard physical activity questionnaires that focus on MET values of activities primarily represent a measure of cardiovascular rather than skeletal load [35]. As bone is known to respond preferentially to quite specific mechanical load parameters, we developed a simple instrument to account for bone-relevant physical activity, the BPAQ. Preliminary data indicates that BPAQ score is predictive of BUA and BMD [25]. Bone geometry is optimally determined using three-dimensional technology, such as computed tomography. For logistical reasons, we used peer-reviewed DXA-derived indices of mechanical characteristics [20] as surrogate geometric measures of bone. While BUA is thought to reflect “bone quality” independent of bone mass and BMD is a two-dimensional measure, neither of them directly captures bone volume, but they are influenced by bone size. To minimise the error associated with this limitation, we reported BMC at each region as recommended by others [36] and used height, weight, and APHV as covariates in statistical comparisons of bone parameters. We also reported BMAD, which effectively adjusts for bone size [19].

## 5. Conclusions

We found that the factors influencing bone status of 13-14-year-old adolescents are sex specific. Maturational status predicts variance in the parameters of bone mass in adolescent girls, while physical activity level and muscle power exert most influence on the bones of adolescent boys. Time spent watching television emerged as a negative influence on bone mass, particularly in boys, an observation with practical implications for health promotion messages. Our findings add to the current relatively modest understanding of the factors influencing the skeletal development of adolescent boys and girls and have implications for the optimal timing of physical activity interventions according to sex in this age group.

## Figures and Tables

**Table 1:** Subject characteristics for adolescent boys and girls (*n* = 99). Mean (SD).

Characteristics	Boys	Girls	*P* value
	*n* = 46	*n* = 53	
Age (years)	13.8 (0.4)	13.7 (0.5)	.16
APHV (years)	13.8 (0.7)	12.2 (0.4)	.001
Weight (kg)	55.8 (2.0)	51.0 (1.2)	.04
Standing height (m)	1.65 (0.01)	1.61 (0.01)	.02
Sitting height (m)	0.85 (0.01)	0.84 (0.01)	.33
BMI (kg·m^−2^)	20.4 (0.6)	19.6 (0.4)	.34
Lean mass (g)	37380 (8390)	30585 (3736)	.001
Fat mass (g)	15983 (8352)	18747 (6239)	.08
% Body fat	22.0 (8.6)	27.7 (5.7)	.002
Vertical jump (cm)	33.6 (1.2)	28.9 (0.9)	.001
Calcium (mg·day^−1^)	1143 (92)	826 (57)	.004
BPAQ score	3.6 (0.2)	3.1 (0.2)	.052
Television viewing (min·day^−1^)	153 (97)	120 (86)	.07

Variable definitions: APHV, age of peak height velocity; BMI, body mass index; BPAQ, bone-specific physical activity questionnaire.

**Table 2:** Bone parameters for adolescent boys and girls (*n* = 99). Mean (SD).

Variables	Boys	Girls	*P* value
	*n* = 46	*n* = 53	
BUA (dB·MHz^−1^)	75.9 (1.9)	81.7 (1.9)	.03
FN area (cm^2^)	4.81 (0.36)	4.46 (0.46)	.001
FN BMC (g)	4.34 (0.14)	3.86 (0.09)	.005
FN BMD (g·cm^−2^)	0.903 (0.025)	0.867 (0.018)	.19
FN BMAD (g·cm^−3^)	0.361 (0.062)	0.364 (0.055)	.80
FN CSMI (cm^4^)	2.98 (0.33)	2.49 (0.70)	.01
TR BMC (g)	9.39 (0.56)	7.17 (0.33)	.001
TR BMD (g·cm^−2^)	0.748 (0.023)	0.674 (0.018)	.01
LS area (cm^2^)	41.1 (5.5)	40.3 (3.8)	.46
LS BMC (g)	32.9 (1.5)	35.4 (1.2)	.15
LS BMD (g·cm^−2^)	0.791 (0.021)	0.875 (0.020)	.006
LS BMAD (g·cm^−3^)	0.116 (0.015)	0.134 (0.019)	.001
LS IBS (g^2^·cm^−4^)	0.813 (0.229)	0.997 (0.280)	.005
WB BMC (g)	2194 (78)	2147 (53)	.66
WB BMD (g·cm^−2^)	0.854 (0.017)	0.849 (0.014)	.88

Variable definitions: BMAD, bone mineral apparent density, BMC, bone mineral content; BMD, bone mineral density; BUA, broadband ultrasound attenuationl; CSMI, cross-sectional moment of inertia; FN, femoral neck; IBS, index of bone structural strength; LS, lumbar spine; WB, whole body.

**Table 3:** Significant relationships between duration of television viewing and bone parameters for adolescent boys and girls (*n* = 99).

Variables	*r* value	*P* value
Boys		
FN Z (cm^3^)	−0.36	.05
TR BMC (g)	−0.44	.01
LS BMC (g)	−0.43	.02
LS BMD (g·cm^−2^)	−0.38	.04
LS IBS (g^2^·cm^−4^)	−0.37	.04
WB BMC (g)	−0.44	.02
WB BMD (g·cm^−2^)	−0.42	.02

Girls		
WB BMC (g)	−0.35	.03

Variable definitions: BMC, bone mineral content; BMD, bonse mineral density; FN, femoral neck; IBS, index of bone structural strength; LS, lumbar spine; TR, trochanter; WB, whole body; Z, index of bending strength.
